# Winging of Scapula: An Uncommon Presentation of a Common Tumor

**DOI:** 10.7759/cureus.12384

**Published:** 2020-12-30

**Authors:** Ashish Rustagi, Anish Agarwalla, Sarang Agarwal, Loveneesh Krishna, Jatin Talwar

**Affiliations:** 1 Orthopaedics, Vardhman Mahavir Medical College and Safdarjung Hospital, New Delhi, IND

**Keywords:** winging of scapula, osteochondroma, uncommon presentation

## Abstract

Neuromuscular causes of winging of scapula are well known, but winging and snapping of scapula may rarely be caused by space-occupying lesion of the thoracic wall. Although osteochondroma of scapula is rare, it is the most common neoplasm of scapula, and osteochondroma of ventral scapula may lead to pseudo-winging, snapping, and rib erosion on the same side. Owing to its rarity, we report two cases of osteochondroma of ventral scapula with complains of difficult scapulothoracic movement (snapping scapula) and pseudo-winging. After initial clinical and radiological investigations, wide local excision was done and diagnosis confirmed histopathologically. In a two-year follow-up, there is no recurrence, and symptoms of snapping and pseudo-winging disappeared completely. Pertaining to its asymptomatic nature and rare location, diagnosis of osteochondroma may be missed initially searching for some other neuromuscular disorders, and these cases should be reported to increase awareness and to execute proper surgical management.

## Introduction

Winging of scapula is mainly caused by neuromuscular factors, with serratus anterior muscle being most commonly involved leading to medial winging and rarely trapezius and rhomboid muscle causing lateral border of scapula to become prominent [1]. Structural abnormalities can also present with winging of scapula and include rotator cuff pathology, shoulder instability, aseptic necrosis of the humeral head, fibrotic shortening of the middle and posterior deltoid, malunited acromial fractures, and acromegalic arthropathy of the shoulder [1]. However, rarely, apart from common neuromuscular causes, either edge of scapula may become prominent due to other reasons and the condition known as pseudo-winging. We elaborate two such cases of pseudo-winging of scapula.

## Case presentation

Case 1: A 17-year-old female presented to the Outpatient Department (OPD) with prominence of left scapula since last one year with mild pain at rest and painful terminal restriction of shoulder movement with a cracking/grinding sound, without any radiating pain in her left arm. The patient had disturbed sleep since past two months and had to lie prone for pain relief.

Case 2: A 10-year-old boy presented to the OPD with complaint of prominence of medial border of left scapula at rest and pain underneath the scapula at rest. Pain was also associated with shoulder movements, with a grinding sound.

**Figure 1:**
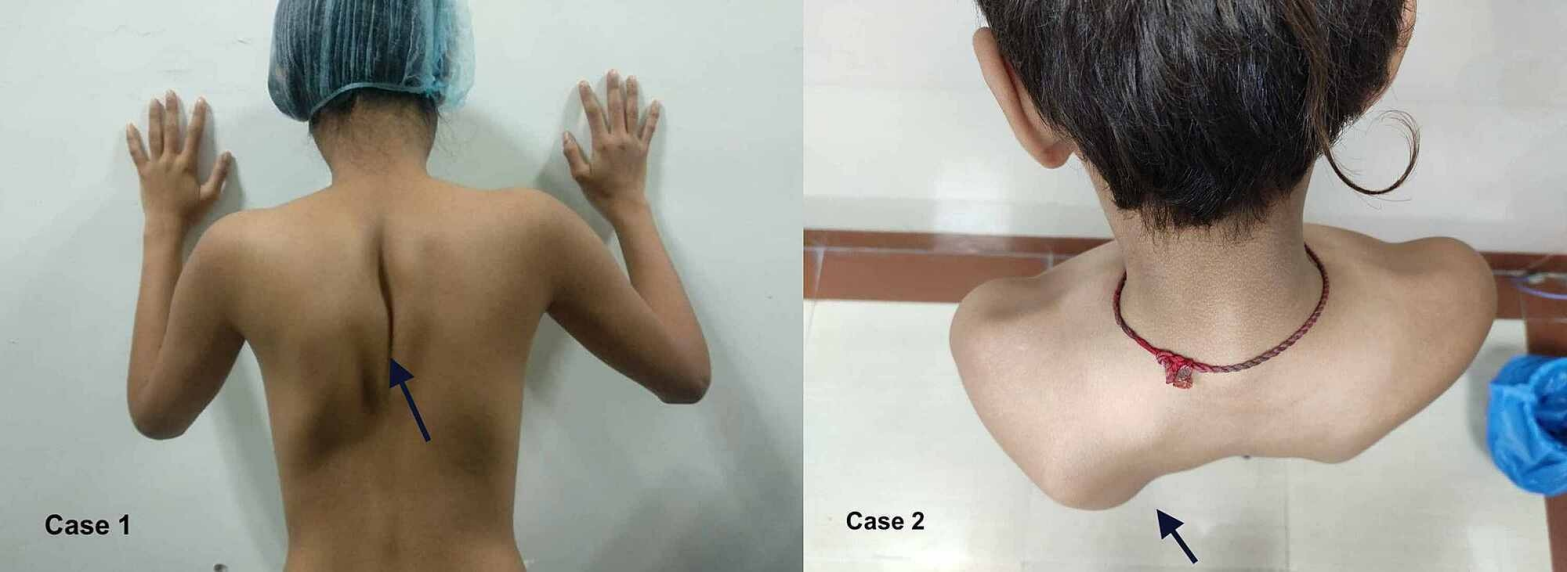
Showing medial prominence of scapula in cases 1 and 2 (black arrows).

Medial edge of scapula was found to be prominent at rest in both the cases, with normal orientation of superior and inferior angles and no changes on forward flexion of arms against resistance (Figure 1).

The patients were evaluated initially for neuromuscular causes, but etiology could not be ascertained to either of the muscle groups. Then on further examination, a swelling was found below the medial edge of scapula, which was missed initially. X-ray was suggestive of a bony lesion arising from the medial edge of scapula. MRI confirmed the provisional diagnosis of osteochondroma, a space-occupying lesion arising from medial and ventral aspect of scapula. Cartilage cap thickness was less than 2 cm in both the cases.

**Figure 2:**
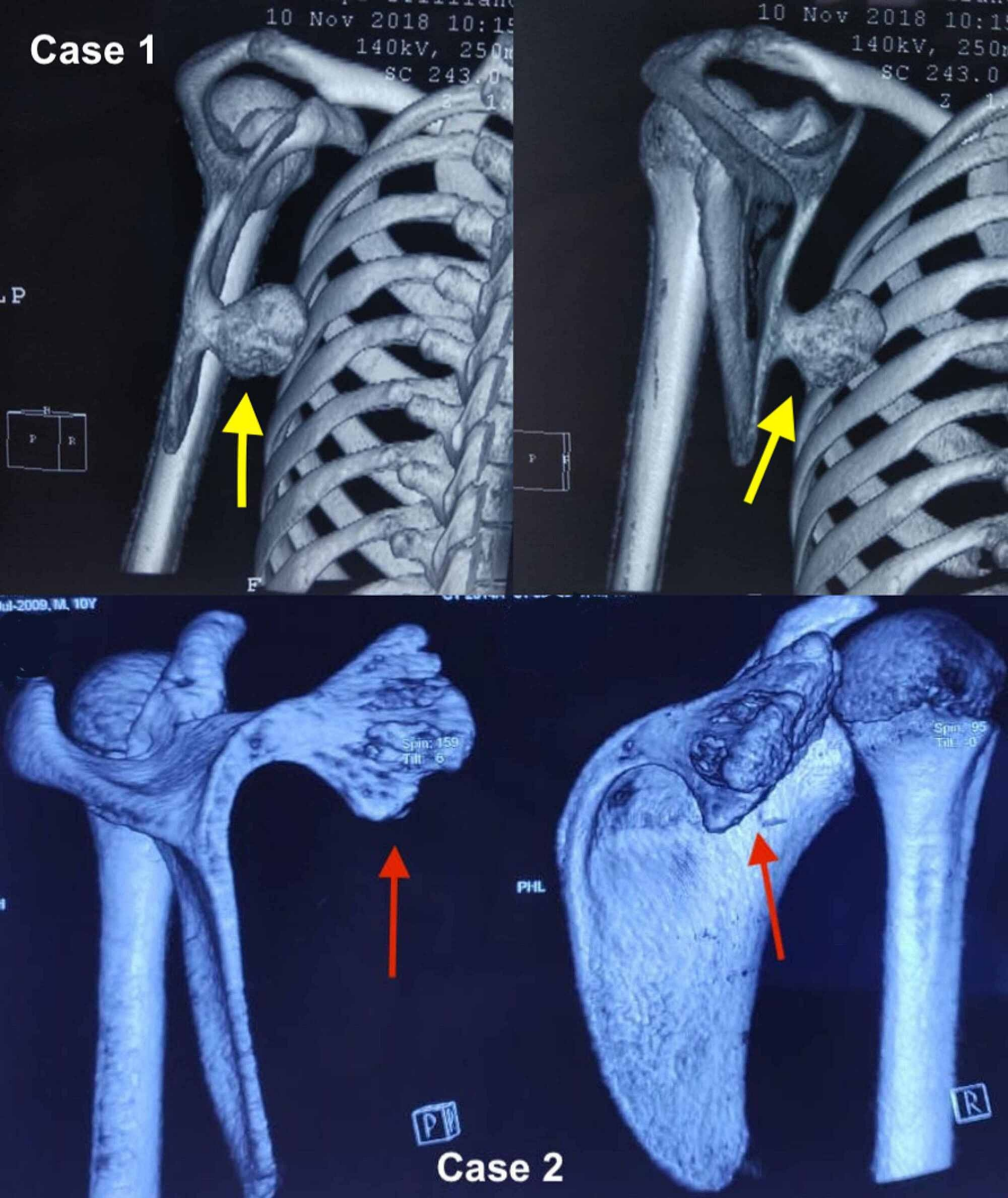
CT scan showing the pedunculated osteochondroma lifting the medial edge of scapula off the thoracic wall in case 1 (yellow arrows) and case 2 (red arrows).

**Figure 3:**
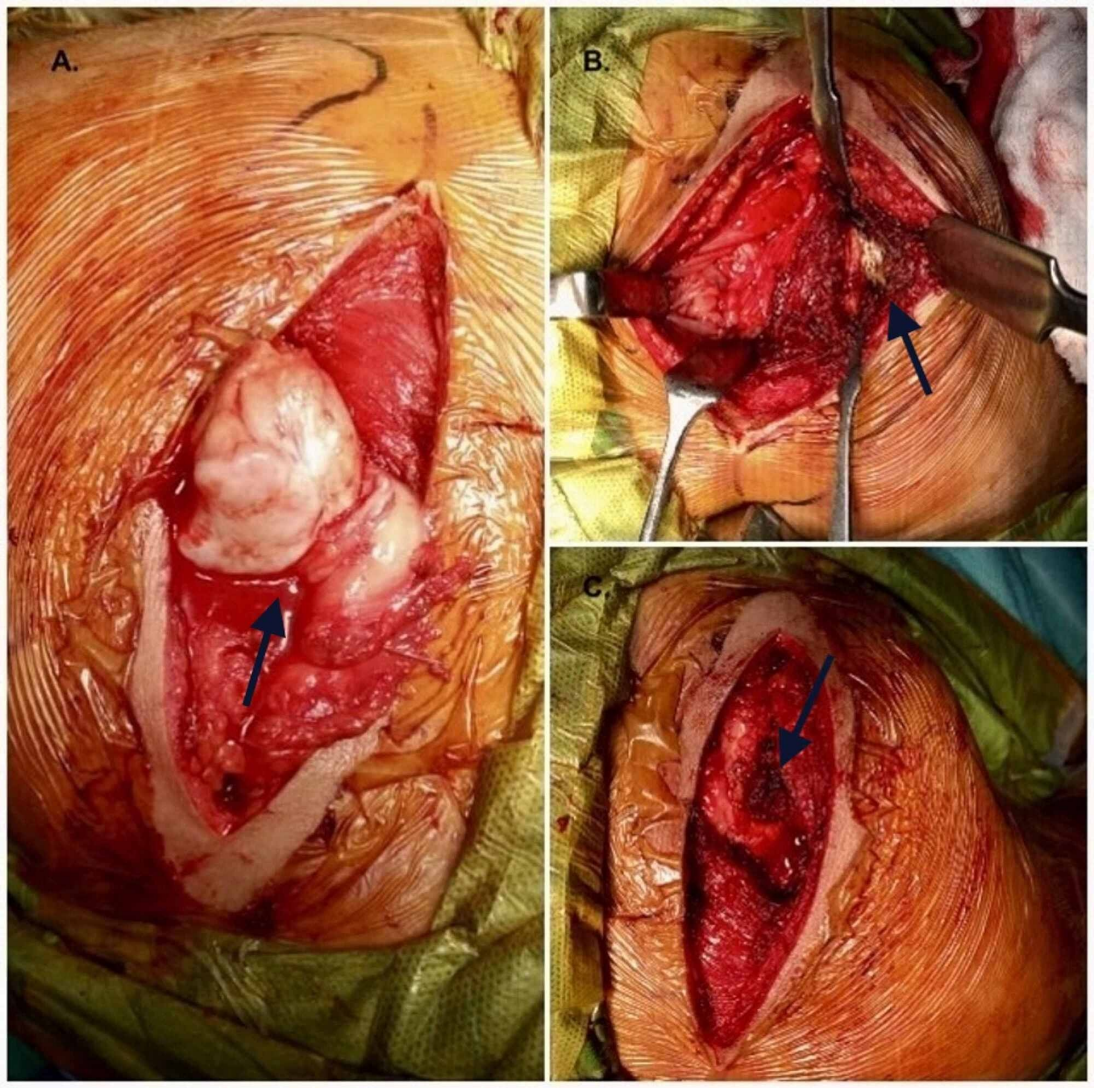
(A) Mushroom-shaped lesion (black arrow), (B) stalk of the lesion (black arrow), and (C) after excision (black arrow).

A pre-operative CT scan (Figure 2) was done, and a pedunculated osteochondroma was seen arising from the ventral aspect of scapula. A wide local excision was planned under general anesthesia. The patients were placed in prone position, and an incision was given 2-3 cm medial to the medial border of scapula. Rhomboid muscle was split along its fibers and retracted to expose the lesion. A large bursa was covering the lesion and excised. After excising the bursa, the scapula retracted to expose the tumor along its whole length (Figure 3).

 

Meticulous dissection was done to avoid injury to thoracic wall and pleura. The lesion was excised extraperiosteally from its base. After excision a dead space was created, and soft tissue was repaired under drain cover to obliterate the dead space.

After the surgery, the patients were monitored in ICU for the suspicion of chest injury, and after ruling out such injury with chest x-ray, the patients were shifted to post-operative ward. Post-operatively, diagnosis was confirmed histologically, and the patients were kept in arm pouch for three weeks, with gentle passive range of motion started as soon as the patients were comfortable and pain-free. Active range of motion and muscle-strengthening exercise were started after two weeks at stitch removal. After two years of follow-up, both patients are doing well without any pain, no signs of recurrence, and full shoulder range of motion.

## Discussion

Winging of scapula is mainly caused by neuromuscular causes, and diagnosis is mainly clinical, sometimes supplemented with MRI cervical spine and electrodiagnostic studies. However, pseudo-winging of scapula is caused by a space-occupying lesion of either ventral scapula or the rib cage posteriorly. Osteochondroma of the scapula causing deformity was first described in 1914. McWilliams described a case of “adventitious bursa” surrounding a scapular exostosis in an 18-year-old female [2]. Osteochondroma can appear in any bone, which develops by endochondral ossification and mainly appears at metaphyseal region of growing long bones and cease to grow after skeletal maturity [3]. Although osteochondroma is the most common benign bone tumor [4], it is rare in flat bones and even rarer in scapula. Osteochondroma is rare in scapula, but it is the most common benign bone tumor of scapula [5]. It is mostly asymptomatic initially and causes symptoms later due to its mass effect on near-by structures [6]. Fiddian and Wing [7] classified the winged scapula according to its mobility as static (A) or dynamic (B), and, per its anatomical origin, in four types: type I, resulting from nervous lesion (most common); type II, related to a muscle lesion; type III, per bone etiology; and type IV, per joint damage. Our patients presented with static medial winging, chronic dull pain, and terminal restriction of shoulder abduction with scapular snapping due to scapula-thoracic movement. Osteochondroma of scapula should be considered as a possible differential diagnosis in a young patient with these symptoms and in the absence of any neuromuscular features [5]. Winging secondary to trapezius muscle paralysis can be diagnosed by physical exam with confirmation by electromyography [1].

It is difficult to diagnose these cases clinically because of its ventral location, and routine chest x-ray may fail to identify the source of origin of the lesion. CT scan helps to accurately map the bony anatomy of the lesion, and MRI can measure the cartilage cap thickness, bursa formation, and rib erosion. Surgical resection of vertical scapular osteotomy (VSO) helps relieve symptoms [8]. Excision can be done either by open means or arthroscopically [9].

Early wide local excision should be considered in these cases because of its cosmetic implications, possible bursitis, and rib erosion, and it also helps in making a confirmatory histopathological diagnosis and preventing recurrence. Orth et al. [10] presented a series of three patients with static winging due to osteochondroma of scapula, which were treated with excision of lesion and reported a good functional outcome.

Other authors have reported success with partial resection of the bursa [11]. Careful surgical dissection is very important to avoid chest wall injury, and soft tissue repair should be done meticulously to avoid dead space formation and possible scapula-thoracic dissociation post-operatively. Review of the literature confirms that in rare cases, scapular osteochondroma of the ventral scapula surface produces pseudo-winging by displacement by bony mass effect or formation of a reactive subscapular bursa and should be considered in the differential diagnosis when winging and a mass are present.

## Conclusions

It is always wise to consider scapular osteochondroma as a cause in a case of static winging of scapula with the symptoms described to avoid unnecessary investigations searching for neuromuscular causes and missing the diagnosis initially.
